# Chemiosmotic Energy Conservation in *Dinoroseobacter shibae*: Proton Translocation Driven by Aerobic Respiration, Denitrification, and Photosynthetic Light Reaction

**DOI:** 10.3389/fmicb.2018.00903

**Published:** 2018-05-09

**Authors:** Christian Kirchhoff, Matthias Ebert, Dieter Jahn, Heribert Cypionka

**Affiliations:** ^1^Institute for Chemistry and Biology of the Marine Environment, Carl von Ossietzky University of Oldenburg, Oldenburg, Germany; ^2^Institute of Microbiology, Braunschweig University of Technology, Braunschweig, Germany

**Keywords:** *Roseobacter* group, aerobic anoxygenic phototrophs, light-harvesting and channeling, *napA*, *nirS*

## Abstract

*Dinoroseobacter shibae* is an aerobic anoxygenic phototroph and able to utilize light energy to support its aerobic energy metabolism. Since the cells can also grow anaerobically with nitrate and nitrite as terminal electron acceptor, we were interested in how the cells profit from photosynthesis during denitrification and what the steps of chemiosmotic energy conservation are. Therefore, we conducted proton translocation experiments and compared O_2_^-^, NO_3_^-^, and NO_2_^-^ respiration during different light regimes and in the dark. We used wild type cells and transposon mutants with knocked-out nitrate- and nitrite- reductase genes (*napA* and *nirS*), as well as a mutant (*ppsR*) impaired in bacteriochlorophyll *a* synthesis. Light had a positive impact on proton translocation, independent of the type of terminal electron acceptor present. In the absence of an electron acceptor, however, light did not stimulate proton translocation. The light-driven add-on to proton translocation was about 1.4 H^+^/e^-^ for O_2_ respiration and about 1.1 H^+^/e^-^ for NO_3_^-^ and NO_2_^-^. We could see that the chemiosmotic energy conservation during aerobic respiration involved proton translocation, mediated by the NADH dehydrogenase, the cytochrome *bc*_1_ complex, and the cytochrome *c* oxidase. During denitrification the last proton translocation step of the electron transport was missing, resulting in a lower H^+^/e^-^ ratio during anoxia. Furthermore, we studied the type of light-harvesting and found that the cells were able to channel light from the green–blue spectrum most efficiently, while red light has only minor impact. This fits well with the depth profiles for *D. shibae* abundance in the ocean and the penetration depth of light with different wavelengths into the water column.

## Introduction

*Dinoroseobacter shibae* is an aerobic anoxygenic phototroph and has become a model organism for the *Roseobacter* group (Buchan et al., 2000; Martens et al., 2006; Wagner-Döbler and Biebl, 2006; Simon et al., 2017). This physiologically heterogeneous group of bacteria is highly abundant in photic zones of marine environments worldwide (Biebl and Wagner-Döbler, 2006). *D. shibae* maintains a fine-tuned regulatory network, which allows for coping with anoxic conditions. It is also capable of using nitrate and nitrite as an alternative electron acceptor (Ebert et al., 2017). All of the genes necessary for the stepwise reduction of nitrate to nitrite (via periplasmic nitrate reductase NapABC), nitric oxide (via nitrite reductase NirS), nitrous oxide (NorBC) and finally dinitrogen (NosZ) were found tightly clustered on the chromosome of *D. shibae* (Wagner-Döbler et al., 2010; Laass et al., 2014).

*Dinoroseobacter shibae* possesses a photosystem containing bacteriochlorophyll *a* and the carotenoid spheroidenone. Although photosynthesis in aerobic anoxygenic phototrophs involves cyclic electron transport only, the presence of a terminal electron acceptor is essential for the utilization of light (Shiba et al., 1979; Harashima et al., 1987; Holert et al., 2011). Under reducing conditions in the absence of terminal electron acceptors, photosynthesis is not performed, as the primary electron acceptor pheophytin is unable to take up electrons.

*Dinoroseobacter shibae* can utilize light energy to quickly regenerate, with the help of an in increased membrane potential, its energetic charge after suffering from short-term anoxia (Holert et al., 2011; Kirchhoff and Cypionka, 2017). Light exposure can also enhance survival of the cells during starvation (Soora and Cypionka, 2013). However, whether *D. shibae* can also benefit from light under anoxic conditions while performing denitrification was unclear.

In this study we wanted to assess the contribution of photosynthesis to chemiosmotic energy conservation during aerobic and anaerobic respiration. For this purpose, we measured the proton translocation under oxic conditions in the light and in the dark and compared this with the same process during denitrification. To examine whether the results are specific for the nitrate- and nitrite- reductase, two insertion mutants (*napA* and *nirS*) were analyzed for their potential in proton translocation (Ebert et al., 2013).

Furthermore, we were interested in the light-harvesting and channeling capabilities of the antenna complex of *D. shibae*. Therefore, we compared the effect of different light colors on proton translocation during aerobic respiration.

## Materials and Methods

### Bacterial Strains and Cultivation

*Dinoroseobacter shibae* DFL 12^T^ (Biebl et al., 2005) was grown in artificial seawater medium (SWM) with 10 mM succinate and 25 mM nitrate in a volume of 150 ml (see Supplement 1). Cells were cultivated anaerobically in a diurnal light/dark rhythm (12 h/12 h, 12 μmol photons m^-2^ s^-1^) in a shaker at 25°C and 125 rpm (Soora and Cypionka, 2013). The expression of the photopigments is inhibited by light and therefore occurs predominantly in the dark (Endres et al., 2015), which makes phototrophy dependent on a day–night cycle, different to anaerobic phototrophs (Harashima et al., 1980; Yurkov and van Gemerden, 1993). The transposon insertion mutants *napA* (DSTn2943) and *nirS* (DSTn3258) (Ebert et al., 2013) lack the nitrate- and nitrite- reductase genes, respectively and are therefore not able to utilize NO_3_^-^ or NO_2_^-^ as terminal electron acceptor. Both were grown within the identical medium as the wild type, but under oxic conditions to provide comparability between both mutants. In control experiments, the *napA* and *nirS* mutants were also successfully grown under anoxic conditions with NO_3_^-^ or NO_2_^-^ as electron acceptor, which resulted in different OD_max_ and generation times. Additionally, the transposon mutant *ppsR* (DSTn4634, Ebert et al., 2013), which does not produce bacteriochlorophyll *a* (see Supplement 2), was used as a control strain.

### Proton Translocation Measurements

**FIGURE 1:**
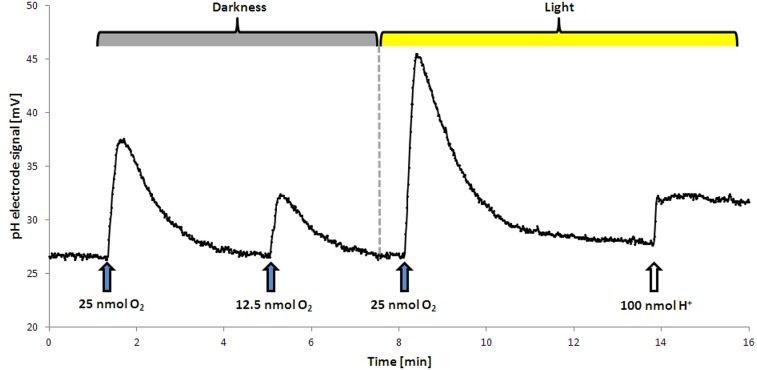
Proton translocation during aerobic respiration in the dark and in the light. Upon addition of O_2_ protons were released into the medium, resulting in a temporary increase in mV. This effect was stronger in the light. The dashed line shows the start point of illumination. No H^+^ translocation upon illumination alone was observed, the addition of a terminal electron acceptor was required. For calibration a defined pulse of HCl was added at the end of the analysis.

The proton translocation experiments were conducted after a modified method after Fitz and Cypionka (1989). Cells were grown for 18 h to an OD_436_ of approximately 0.8 and subsequently harvested by centrifugation (150 ml culture, 10,000 ×*g*, 10 min, 4°C, Beckman J2-HS). The supernatant was discarded and the pellet was resuspended in 6 ml of non-buffered solution (300 mM NaCl, pH 7.4) and stored on ice. Proton translocation measurements were performed in a small reaction tube (3 ml) at 30°C. The tube was filled with 2 ml of cell suspension, 200 μl of KSCN (1 M in H_2_O) and 10 μl succinate (500 mM in H_2_O). Afterward the tube was closed with a rubber stopper and constantly mixed with a magnetic stirrer. The stopper had an in- and out-let for the N_2_-gassing to provide constant anoxic conditions, an opening for the pH electrode (type Inlab Micro, Mettler Toledo) and for the addition of various electron acceptors. For the establishment of anoxic conditions, the suspension was gassed for 30 min with N_2_. This gives the cell enough time to establish the denitrification apparatus. During subsequent steps the anoxic conditions were kept by continuous N_2_-flushing. Small amounts of O_2_ (10–20 μl O_2_-saturated H_2_O, 12.5–25 nmol), NO_3_^-^ (10–20 μl 1 mM NO_3_^-^ in H_2_O, 10–20 nmol) NO_2_^-^ (10–20 μl 1 mM NO_3_^-^ in H_2_O, 10–20 nmol) were added to the suspension and pH changes were recorded (**Figure 1**). For this purpose, a pH electrode was connected to an AD converter (ADC-16, pico Technology) which was handled by software (MPwin version 2008.08.25, Cypionka, 2005). KSCN was added to decrease the membrane potential of the cells by slowing down the backflow of the protons along the electrochemical gradient. The observed changes in pH were compared to calibration pulses (10 μl of 10 mM HCl) at the end of each experiment (**Figure 1**). This allowed for the calculation of the amount of protons translocated per added electron acceptor. Each value was extrapolated back to the time of electron acceptor addition, which usually resulted in an increase of about 10%. Exemplary proton translocation experiments with NO_3_^-^ or NO_2_^-^ can be found in Supplement 3.

### Set-Up for Light Quality Studies

To compare the proton translocation under different light conditions, we constructed a light cabinet with an interior coated by white paper. A lid allowed the operation of experiments from the outside. The cabinet held three types of LEDs (type 18418 1–3, Barthelme, Germany, 628, 515, and 476 nm), which allowed for the adjustment of defined conditions of red, green, and blue light. The intensity was set to 9.6 μmol photons m^-2^ s^-1^ with the help of a dimmer (Light meter model LI-189 by LI-COR, United States) For white light, all three LED types were activated simultaneously, with an intensity of 9.6 μmol photons m^-2^ s^-1^ each. In pre-experiments this intensity was shown to be non-saturating for a cell suspension of OD_436_ 25.

## Results

### Light Supports Proton Translocation Independent of the Terminal Electron Acceptor of Respiration

**FIGURE 2:**
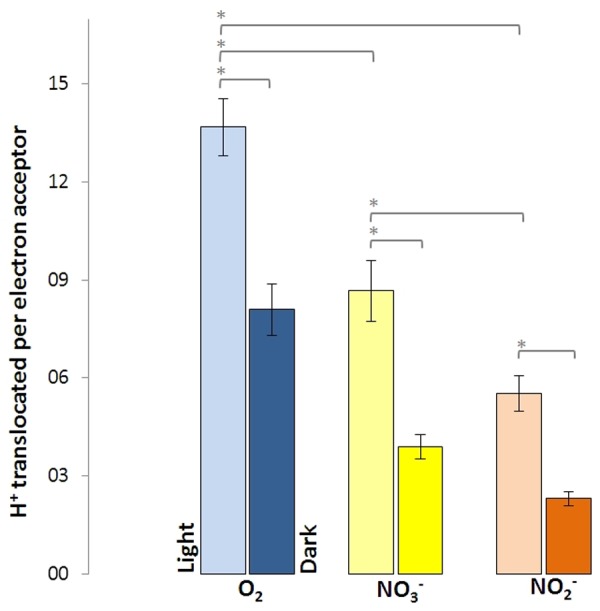
Protons translocated per electron acceptor molecule measured in white light and in the dark for *Dinoroseobacter shibae* wild type cells. Utilization of O_2_ as electron acceptor resulted in the most protons translocated, followed by NO_3_^-^ and NO_2_^-^_._ Error bars indicate standard errors. *P*-values were determined using ANOVA and *post hoc t*-test. Asterisk indicates *P*-values ≤ 0.05. *n* = 6–8.

Washed cells of *D. shibae*, previously grown aerobically with succinate in the presence of nitrate, were able to utilize O_2_, NO_3_^-^, and NO_2_^-^ as terminal electron acceptors, when tested in an unbuffered cell suspension after harvesting. The quantitative measurement of proton translocation required the addition of KSCN to the suspension in order to lower the membrane potential of the cells and slow down the backflow of protons. The cells were able to translocate protons in the light and in the dark, although the amount of measured translocated protons per electron acceptor was different (**Figure 2**). In the dark, 8.1 H^+^ were translocated per O_2_ (mol/mol), followed by 3.9 H^+^ per NO_3_^-^ and 2.3 H^+^ per NO_2_^-^. In the light, these values increased significantly for all tested electron acceptors, with O_2_ now allowing for the translocation of 13.7 H^+^, followed by 8.7 H^+^ per NO_3_^-^ and 5.5 H^+^ per NO_2_^-^.

**FIGURE 3:**
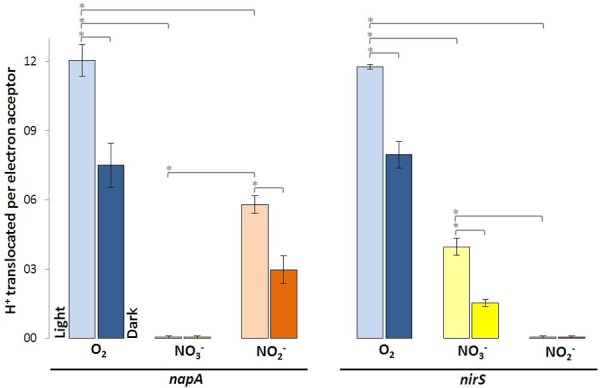
Protons translocated per electron acceptor molecule determined in white light and in the dark using indicated *D. shibae* transposon mutants. Error bars indicate standard errors. *P*-values were determined using ANOVA and *post hoc t*-test. Asterisk indicates *P*-values ≤ 0.05. *n* = 5–6.

The nitrate reductase-deficient mutant *napA* and the nitrite-deficient mutant *nirS* translocated more protons per molecule O_2_ than with NO_3_^-^ and NO_2_^-^. For the *napA* mutant strain almost no proton translocation was observed upon addition of NO_3_^-^. O_2_^-^ and NO_2_^-^ respiration were still operative in the *napA* mutant and significantly more H^+^ were translocated in the light than in the dark (**Figure 3**, left). Vice versa, the *nirS* mutant strain did not translocate H^+^ upon NO_2_^-^ addition, while O_2_^-^ and NO_3_^-^ respiration was still operative (**Figure 3**, right). However, this time less H^+^ were translocated upon NO_3_^-^ addition compared to the wild type and *napA* strain. This is likely due to the accumulation of NO_2_^-^.

### Green Light Enhances Proton Translocation Most Efficiently, While Red Light Has Only Minimal Impact

**FIGURE 4:**
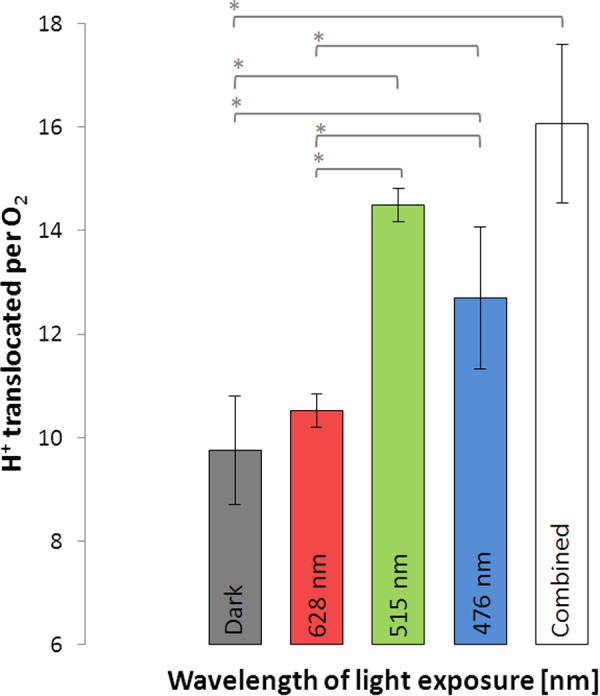
Protons translocated per molecule O_2_ during illumination with light of different wavelengths and in the dark using *D. shibae* wild type. Error bars indicate standard errors. *P*-values were determined using ANOVA and *post hoc t*-test. Asterisk indicates *P*-values ≤ 0.05. *n* = 6.

Light-driven proton translocation depended on the applied wavelength. Red light (10.5 ± 0.3) had only little impact on the H^+^/O_2_ ratio, while green and blue light significantly enhanced the H^+^/O_2_ ratio (**Figure 4**). In the green light (14.5 ± 0.3) the cells translocated slightly more protons than in the blue light (12.7 ± 1.4). The bacteriochlorophyll *a*-deficient *D. shibae ppsR* mutant did not show any increase in proton translocation in the light at all tested wavelengths (see Supplement 4).

## Discussion

In the present study, we have assessed proton translocation driven by aerobic respiration and denitrification with- and without illumination. We were able to differentiate the steps of chemiosmotic energy conservation in the electron transport chain of *D. shibae* and determined the influence of light of different wavelengths on them.

### Light Supports Aerobic and Anaerobic Proton Translocation of *D. shibae*

**FIGURE 5:**
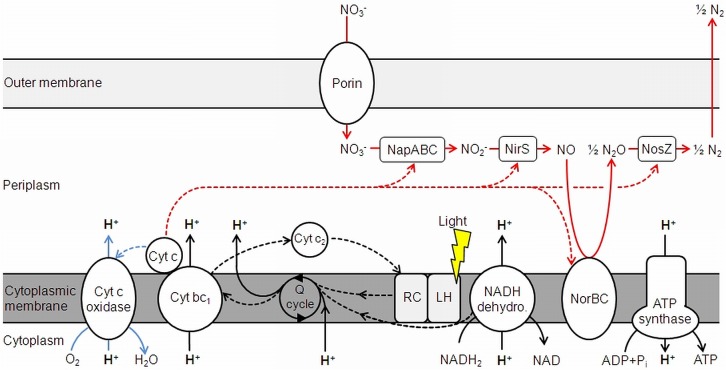
Electron transduction (dashed lines) and proton translocation in *D. shibae*. Denitrification is shown in red, aerobic respiration in blue. NapABC, nitrate reductase; NirS, nitrite reductase; NorBC, nitric oxide reductase; NosZ, nitrous oxide reductase; Cyt, cytochrome; Q cycle, quinone cycle. Modified and extended after Wagner-Döbler et al. (2010) and Laass et al. (2014).

As *D. shibae* is an aerobic anoxygenic phototrophic organism the photosystem can only support proton translocation when a terminal electron acceptor is present. This was confirmed in control experiments (**Figure 1**). Our observations suggested that light adds always the same surplus to the proton translocation during respiratory electron transduction, independent of the electron acceptor added. Apparently, the photosystem of *D. shibae* channels electrons into the electron transport chain for aerobic and anaerobic respiration in a comparative manner. This might be achieved by the reduction of the ubiquinone pool, as it was described for closely related purple bacteria (Klamt et al., 2008) and other Alphaproteobacteria (Wagner-Döbler and Biebl, 2006). The presence of cytochrome *c*_2_ genes in the genome of *D. shibae* (Wagner-Döbler et al., 2010) indicates a cyclic, light-driven electron transport between cytochrome *c_2_* and the cytochrome *bc*_1_ complex (**Figure 5**), which might also work in the same way during denitrification. When no electron acceptor is available and the electron transport chain is fully reduced, the cyclic electron transport cannot proceed.

It has to be taken into account that the measured proton translocation values are possibly underestimated, since the whole system is affected by the depression of the membrane potential by the addition KSCN to slow down the proton reflux (see section “Materials and Methods”). This might affect the H^+^/e^-^ ratios. Anyhow, the differences in proton translocation between O_2_, NO_3_^-^, and NO_2_^-^ can still be clearly distinguished.

When the results of the proton translocation were calculated as H^+^/e^-^ ratio, O_2_ respiration fall from about 3.5 H^+^/e^-^ in the light to 2.1 H^+^/e^-^ in the dark, while both NO_3_^-^ and NO_2_^-^ were reduced from 1.9 to 0.8. It is interesting that the H^+^/e^-^ ratio of both NO_3_^-^ and NO_2_^-^ were equal, although both are electron acceptors of different redox potential. This might be explained by the fact that both NapABC and NirS are not proton pumping. Still, the proportion between light and dark, as well as between O_2_, NO_3_^-^, and NO_2_^-^ is reliable. This is on the basis that the reduction of O_2_ to H_2_O takes up 4 e^-^ and the complete reduction of NO_3_^-^ to N_2_ takes up 5 e^-^.

At a non-saturating intensity, light supported the proton translocation during aerobic respiration for 1.4 H^+^/e^-^ and for 1.1 H^+^/e^-^ during denitrification. Light generates a cyclic electron transport, while respiration relies on a linear electron transport. Consequently, a stoichiometric coupling between respiration-driven and light-driven proton translocation cannot be expected. Considering, that the respiratory rate decreases during illumination (Holert et al., 2011), the impact of light on proton translocation can be considered even higher than observed. The overall excess in translocated protons during aerobic respiration compared to denitrification, regardless of light exposure, leads to the conclusion that this is the result of the cytochrome *c*-oxidase, translocating additional protons across the membrane. This step only occurs in aerobic respiration, since O_2_ is reduced to H_2_O in the process (**Figure 5**). As outlined above, NapABC and NirS are not contributing to the proton gradient.

The steps of chemiosmotic energy conservation via translocated protons across the membrane during aerobic and anaerobic respiration are the NADH-dehydrogenase and the cytochrome *bc*_1_ complex. The succinate dehydrogenase also channels electrons into the Q cycle, but does not directly translocate H^+^ and is therefore excluded from **Figure 5**. The cytochrome *c* oxidase also adds to proton translocation, but only during aerobic respiration.

### Nitrate Reduction Is Impaired When the Nitrite Cannot Be Further Metabolized

The transposon insertion mutants *napA* and *nirS* demonstrate the essential function of NapABC and NirS for the terminal reduction steps of the denitrification pathway. In the nitrite reductase deficient mutant *nirS* NO_2_^-^ is accumulating. This obviously affects further nitrate reduction after short time, since the reaction balance is disturbed. Hence, the decrease in proton translocation compared to the wild type and the *napA* mutant strain can be explained (**Figure 3**, right).

### Channeling of Blue–Green Light Is Reflected in the Environmental Distribution

Putting our results into context with the *in vivo* absorption spectrum of *D. shibae* (Biebl and Wagner-Döbler, 2006), the observed action spectrum of proton translocation efficiency (green > blue > > red) fits exactly the original data. It is build up by the combined absorption of bacteriochlorophyll *a* and the carotenoid spheroidenone within the light harvesting complex of *D. shibae* (Yurkov and Beatty, 1998; Biebl et al., 2005; Niedzwiedzki et al., 2017). Photon capturing by the present pigments and subsequent channeling into the reaction center was most efficient for light of the blue–green part of the spectrum and almost missing for red light. The adaptation of *D. shibae* to green–blue light is an adaptation to the highest penetration depth in the ocean. This is reflected by the depth profiles of aerobic anoxygenic phototroph abundance, which decreases from near sea surface to approximately 150 m depths (Ritchie and Johnson, 2012), following the penetration depths of preferred wavelengths of light.

## Conclusion

The present study demonstrates the impact of light on the chemiosmotic energy conservation of *D. shibae*. It was documented that **(1)** proton translocation for both aerobic and anaerobic respiration is supported the almost identical degree by a light-driven cyclic electron transport. Furthermore, we have **(2)** identified the steps involved in proton translocation at the cytoplasmatic membrane for both types of respiration in darkness and light. Additionally, it turned out that **(3)** the light-harvesting antenna complex of *D. shibae* effectively captures photons from the blue–green spectrum to support proton translocation, while red light is almost not utilized. This fits with the environmental abundance of aerobic anoxygenic phototroph within the water column.

## Author Contributions

All authors listed have made a substantial, direct and intellectual contribution to the work, and approved it for publication.

## Conflict of Interest Statement

The authors declare that the research was conducted in the absence of any commercial or financial relationships that could be construed as a potential conflict of interest.
