# Supplementary material for: Chemiosmotic Energy Conservation in Dinoroseobacter shibae: Proton Translocation Driven by Aerobic Respiration, Denitrification, and Photosynthetic Light Reaction
Source: Front Microbiol. 2018 May 9;9:903. doi: 10.3389/fmicb.2018.00903 (PMC5954134; doi:10.3389/fmicb.2018.00903)
Supplement: Supplementary file 1 [file Data_Sheet_1.DOCX]

**Supplementary material**

**Chemiosmotic energy conservation in *D. shibae*: Proton translocation driven by aerobic respiration, denitrification and photosynthetic light reaction**

**Supplement 1 – Growth media**

Artifical seawater medium (SWM):

SWM-basis (5x):

NaCl 100 g

Na_2_SO_4_ 20 g

MgCl_2_ · 6 H_2_O 15 g

KCl 2.5 g

NH_4_Cl 1.25 g

KH_2_PO_4_ 1 g

CaCl_2_ · 2H_2_O 0.75 g

Dissolve in 800 mL dH_2_O, then bring the volume to 1000 mL with dH_2_O and autoclave for 20 min at 121°C.

NaHCO_3_ stock solution (100x):

1.9 g NaHCO_3_

Dissolve in 100 ml dest. H_2_O and autoclave for 20 min at 121°C.

0.5 M Succinate solution (100x):

Dissolve 29.52 g succinic acid (M = 118.09 g/mol) in 400 ml dH_2_O. Adjust pH value with NaOH to pH 7.5. Bring to the volume of 500 ml with dH_2_O and autoclave for 20 min at 121°C.

| Trace element solution (1000 ml, 1000x): | |  |
| --- | --- | --- |
| H_2_O | 50 ml | |
| FeSO_4_ · 7H_2_O | 2.1 g | |
| 25% HCl | 13 ml | |
| Titriplex III (Na_2_EDTA) | 5.2 g | |
| H_3_BO_3_ | 30 mg | |
| MnCl_2_ · 4H_2_O | 100 mg | |
| CoCl_2_ · 6H_2_O | 190 mg | |
| NiCl_2_ · 6H_2_O | 24 mg | |
| CuCl_2_ · 2H_2_O | 2 mg | |
| ZnSO_4_ · 7H_2_O | 144 mg | |
| Na_2_MoO_4_ · 2H_2_O | 36 mg | |

Autoclave for 15 min at 121°C

Vitamin stock solution (100x):

Biotin 2 mg

Nicotinic acid 20 mg

4-aminobenoic acid 8 mg

Dissolve in 100 mL dest. H_2_O and filter-sterilize (0.2 µm pore size).

To obtain 100 ml SWM 10 mM succinate, compound the stock solutions as follows:

H_2_O, dest, autoclaved 73.9 ml

SWM-Basis 20 ml

Succinate stock 2 ml

NaHCO_3_ stock 3 ml

Vitamin stock solution 1 ml

Trace element solution 100 µl

Set the pH to 7.5 with HCl and KOH. The optimal growth range for *D. shibae* strain DFL 12^T^ is between 6.5 and 8.8 (Biebl *et al.* 2005).

Lysogeny broth (LB medium):

Bacto trypton 10.0 g

Bacto yeast extract 5.0 g

NaCl 10.0 g

Dissolve in 800 mL dH_2_O, set the pH to 7.0 with HCl and KOH, then bring the volume to 1000 ml. Autoclave for 20 min at 121°C. For agar plates add 12 g agar.

Literature

Biebl, H., Allgaier, M., Tindall, B. J., Koblizek, M., Lünsdorf, H., Pukall, R. and Wagner-Döbler, I. (2005). *Dinoroseobacter shibae* gen. nov., sp. nov., a new aerobic phototrophic bacterium isolated from dinoflagellates. *Int. J. Syst. Evol. Microbiol*. 55, 1089–1096. doi: 10.1099/ijs.0.63511-0

**
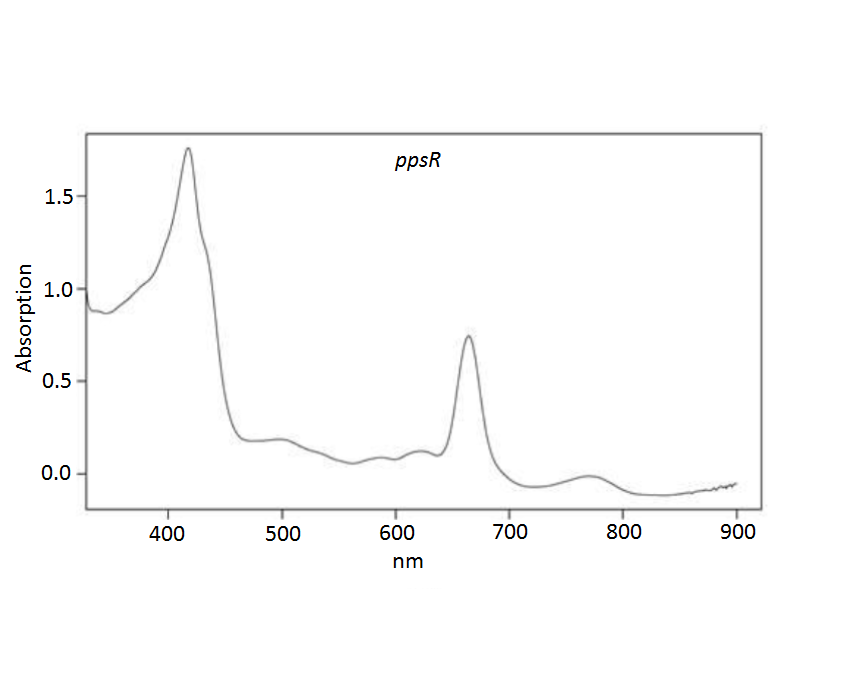
Supplement 2 – Absorption spectra of *ppsR* mutant and wild type**

**
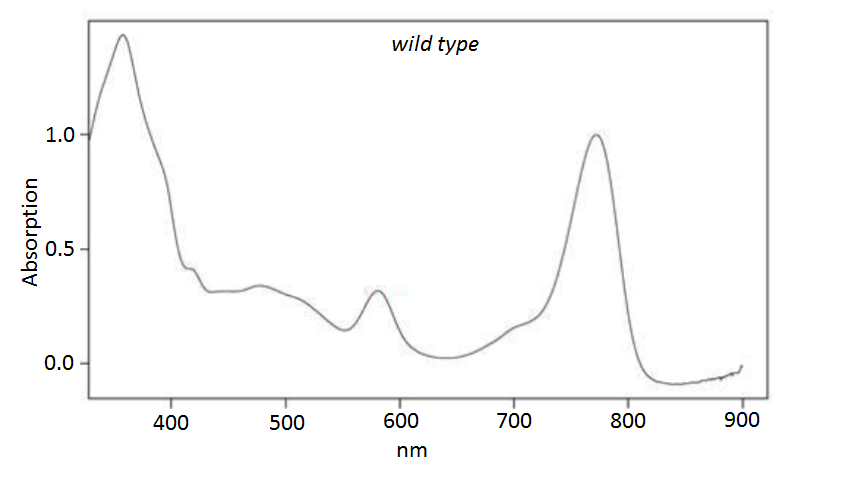
**

Fig. 2 – Absorption spectra of extracted pigments from the *ppsR* mutant and the wild type of *D. shibae*. While the *ppsR* mutant does not have the bacteriochlorophyll-characteristic peak at 780 nm, the wild type still has it. Therefore, we assume that the *ppsR* mutant does not contain bacteriochlorophyll. The peak at 665 nm is likely the result of an accumulation of chlorophyllide, a precursor for bacteriochlorophyll synthesis.

**Extraction protocol for bacteriochlorophyll**

- Harvest of cell culture at OD_578_ 2.5
- 15 ml culture in falcon tube
- Centrifugation for 10min at 4000 rpm, 4°C
- Discard supernatant
- Resuspend pellet in 1 ml acetone/methanol (7/2) for 1 h in darkness on a shaker
- Centrifugation for 5 min at 4000 rpm, 4°C
- Transfer supernatant in reaction tube
- Measurement of absorption spectra 300 – 900 nm
  - Acetone/methanol (7/2) as blank
  - Quartz glass cuvette (d=10 mm)

**Supplement 3 – Proton translocation during denitrification in the dark and in the light**

**
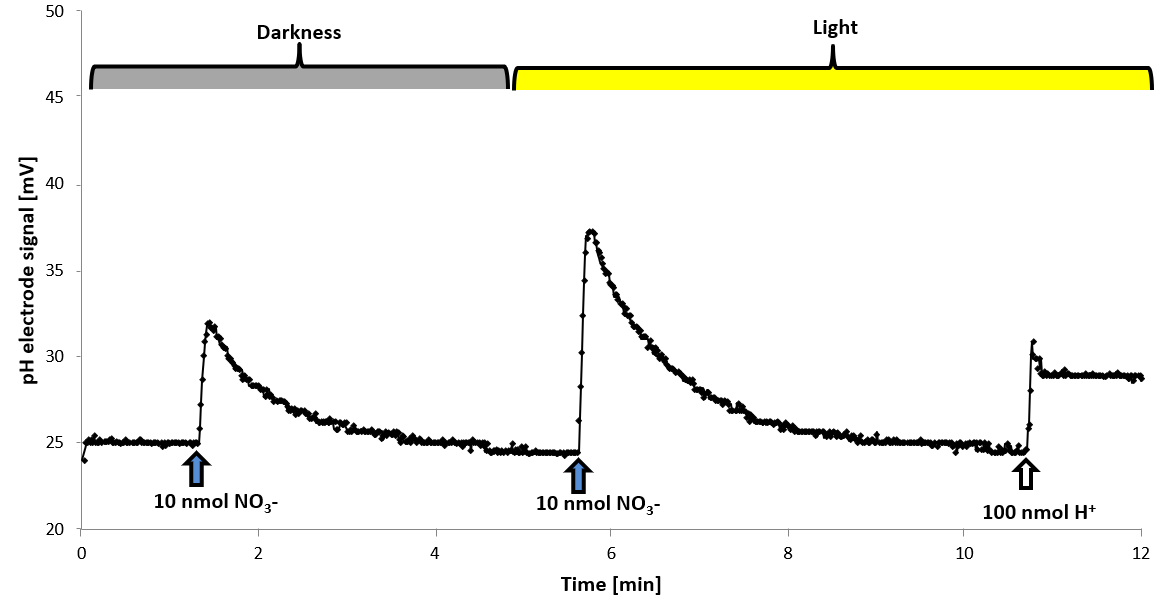
**

**
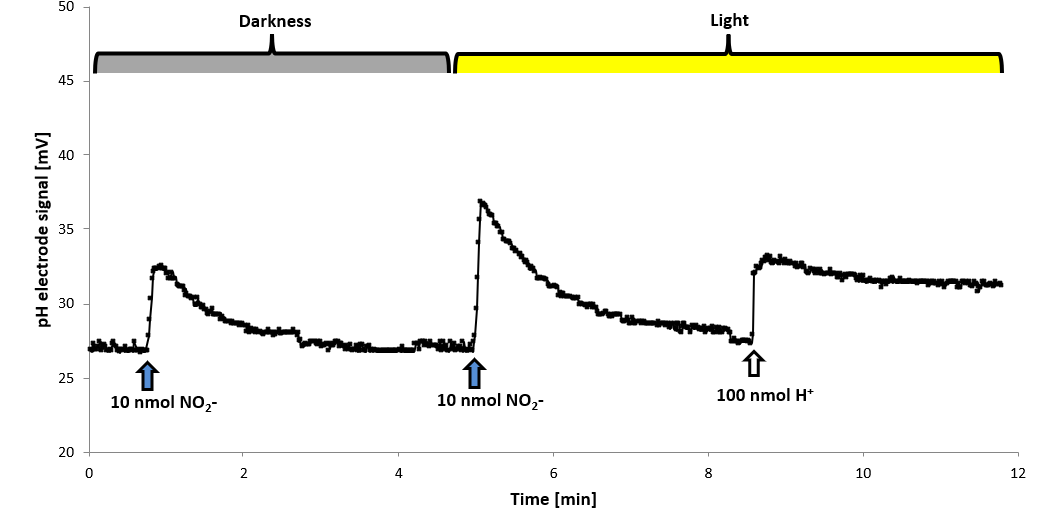
**

Fig. 3 – Proton translocation during denitrification in the dark and in the light. Upon addition of NO_3_^-^ and NO_2_^-^ protons were released into the medium, resulting in a temporary increase in mV. This effect was stronger in the light. For calibration a defined pulse of HCl was added at the end of the analysis.

**Supplement 4 – Proton translocation for *ppsR* mutant at different wavelengths**


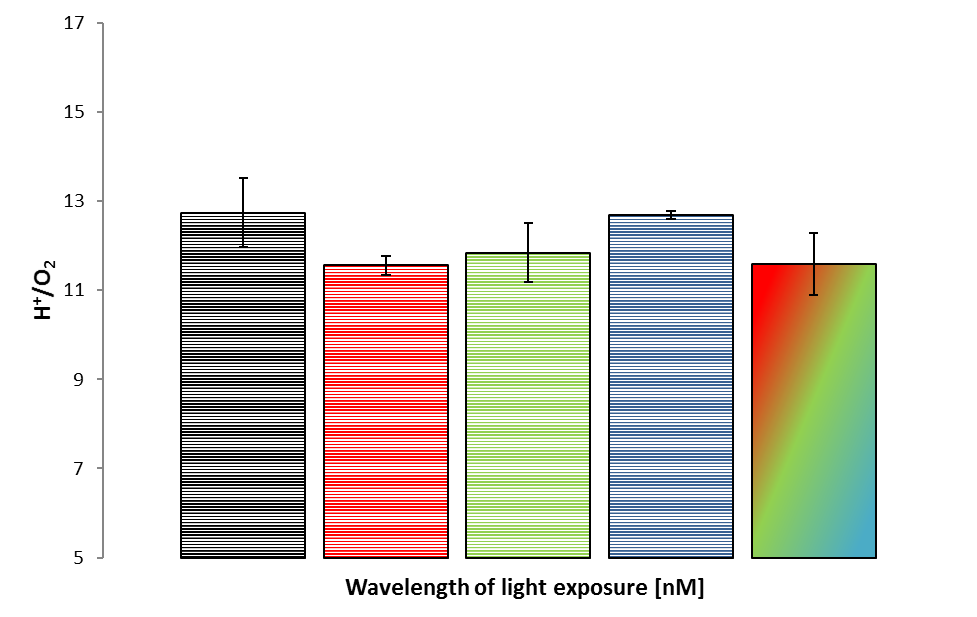


**Darkness**

**628 nm**

**515 nm**

**476 nm**

**Combined**

Fig. 1 – *D. shibae* *ppsR*: Protons translocated per O_2_ during light of different wavelengths and in the dark. Error bars indicate standard errors. P-values were determined using ANOVA and post-hoc t-test. Asterisk indicates P-values ≤ 0.05.
